# Supplementary material for: Thermal excitation signals in the inhomogeneous warm dense electron gas
Source: Sci Rep. 2022 Jan 20;12:1093. doi: 10.1038/s41598-022-05034-z (PMC8776784; doi:10.1038/s41598-022-05034-z)
Supplement: Supplementary file 1 — Supplementary Information. [file 41598_2022_5034_MOESM1_ESM.pdf]

Supplementary Information: Thermal Excitation Signals in the Inhomogeneous Warm Dense Electron Gas

Zh. A. Moldabekov,[1,2] T. Dornheim,[1,2] and A. Cangi[1,2,*]
[1] *Center for Advanced Systems Understanding (CASUS), D-02826 Görlitz, Germany*
[2] *Helmholtz-Zentrum Dresden-Rossendorf, D-01328 Dresden, Germany*

FINITE-SIZE EFFECTS

A potential source of systematic errors are so-called finite-size effects, i.e., the difference between the results for N electrons in a finite simulation cell of volume $V = L^3$ and the thermodynamic limit ($N \to \infty$ with $n = N/V = \text{const}$). Recently it was shown in Ref. [1] that KS-DFT simulations results for the harmonically perturbed electron gas with $N = 14$ is not affected by finite-size effects in both weak and strong perturbation cases. Moreover, it is known from previous studies of the warm dense electron gas that the electronic density response is remarkably well converged with respect to N at the present parameters for as few as $N = 14$ electrons; see, e.g., Refs. [2, 3] and Refs. [4, 5] for corresponding investigations in the linear and nonlinear response regimes, respectively. For the parameters considered in this paper, this is demonstrated in Fig. 1, where the static response functions for $N = 14$ electrons and for $N = 34$ electrons in the unit cell are compared at $q \lesssim 1.5\ q_F$. Furthermore, Dornheim and Vorberger have recently shown [6] that the same is also true for the dynamic structure factor $S(\mathbf{q}, \omega)$. Additionally, in Fig. 2 we show the EELF at increasing N for a periodically inhomogeneous electron gas with the perturbation given in Eq. (1) of the main text. Due to the discretization, a given N corresponds to the wave number $Q = 4\pi/L$ (with $L \sim N^{-1/3}$) of the external perturbation. This complicates the comparison. Nevertheless, in Fig. 2 we illustrate the EELF for wave numbers that are close to each other. In particular, we observe that the new features in Fig. 2 for different N are quite similar and that the difference is primarily due to the different values of Q.

For completeness, we note that the absence of finite-size effects does generally not hold for integrated quantities like the interaction energy per particle v and the related XC energy per particle e_{XC} Yet, it is well-known that these finite-size effects are predominantly due to the approximation of a continuous integral by a sum over discrete $\mathbf{q}$-vectors in the finite simulation cell [7–9] and *not* due to an intrinsic N-dependence in the static structure factor or the density response function themselves. In practice, our DFT simulations employ an LDA XC functional that has been obtained within the TDL to simulate a finite number of electrons N, which appears somewhat inconsistent. On the other hand, the comparison between DFT and the exact QMC solution for finite N in Fig. 3 in the main text reveals excellent agreement both for weak and strong degrees of inhomogeneity.

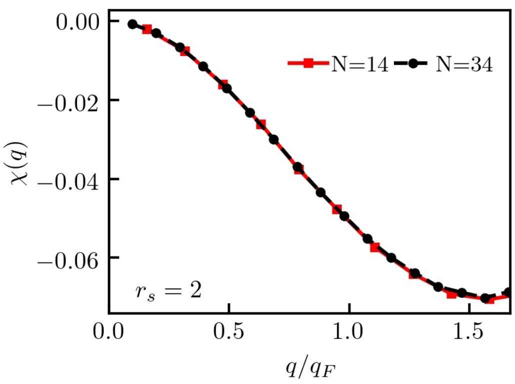

FIG. 1. Static response function of the homogeneous electron gas at $r_s = 2$ and $T = 0.125$ eV from KS-DFT calculations within the adiabatic local density approximation for different numbers of particles in a unit cell.

We, therefore, conclude that finite-size effects do not play an important role in the present study both with respect to the density response and with respect to the XC functional in DFT.

FURTHER DETAILS ON PLASMON-LIKE MODES IN THE EELF SPECTRUM

Here, we provide additional details on the plasmon-like modes that appear in spatially inhomogeneous WDM, while we focus on the emergence of unexplored modes and features in the main text. We provide supporting evidence on the behaviour of these plasmon-like modes, as they do not show a pronounced dependence on temperature. Therefore, these modes might be utilized as a gauge when a temperature diagnostics of WDM is performed based on the unexplored, temperature-sensitive modes.

These plasmon-like modes of inhomogeneous WDM appear in different directions with respect to the perturbation $\mathbf{Q}$. For example, they appear in the transverse direction to $\mathbf{Q}$. They also appear at angles towards $\mathbf{Q}$, for instance in the $\mathbf{q}_{\text{xy}}$ and $\mathbf{q}_{\text{yz}}$ planes. The properties of these modes and their similarity to standard plasmons within homogeneous WDM are discussed below.

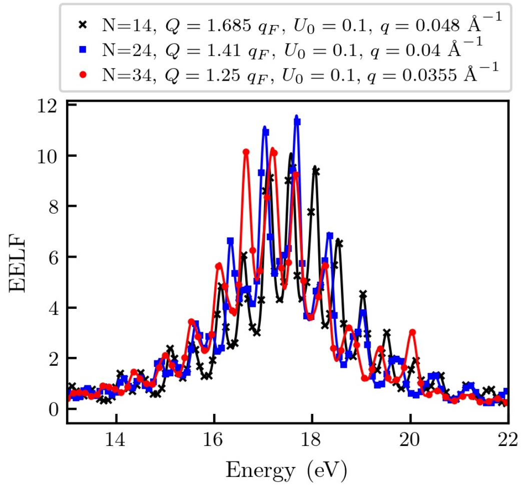

FIG. 2. The EELF of an inhomogeneous electron gas computed from KS-DFT calculations for different numbers of particles in a unit cell at $r_s = 2$ and $T = 1.17$eV.

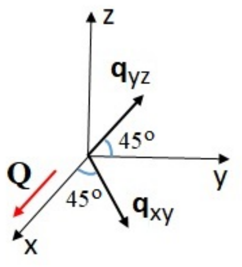

FIG. 3. Directions in which the inhomogeneous perturbation of WDM is probed. The direction of the perturbation is denoted by the wave vector $\mathbf{Q}$. Two momentum transfer wave vectors ($\mathbf{q}_{\rm xy}$ and $\mathbf{q}_{\rm yz}$) are also shown.

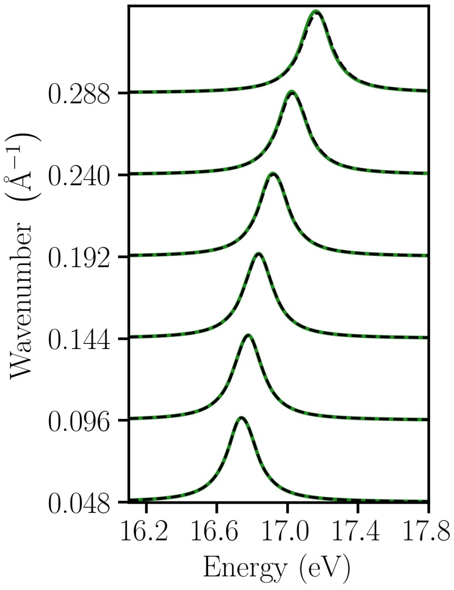

FIG. 4. The EELF at $\theta = 0.5$ and $U_0 = 0.1$. The dashed black curve corresponds to the case of homogeneous system and solid green curve is for the case of inhomogeneous system with $\mathbf{q}$ in transverse to $\mathbf{Q}$ direction (along z axis).

The directions of vectors $\mathbf{Q}$, $\mathbf{q}_{\rm xy}$, and $\mathbf{q}_{\rm yz}$ are illustrated in Fig. 3.

In the transverse direction to the perturbation

First, we consider plasmon-like modes that emerge in the transverse direction to $\mathbf{Q}$. Results at different perturbation amplitudes and temperatures are shown in Figs. 4-7. In Fig. 4 we compare the EELF transverse to $\mathbf{Q}$ at a perturbation amplitude $U_0 = 0.1$ and $\theta = 0.5$ with the plasmon mode of the homogeneous system. In Fig. 5 we illustrate the temperature dependence of that mode. In Fig. 6 and Fig. 7 we repeat this analysis at a perturbation amplitude $U_0 = 1.0$. From these we conclude that the EELF of the inhomogeneous system probed in the transverse direction to $\mathbf{Q}$ does not exhibit a sensitivity towards temperature in the limit $q \to 0$. However,

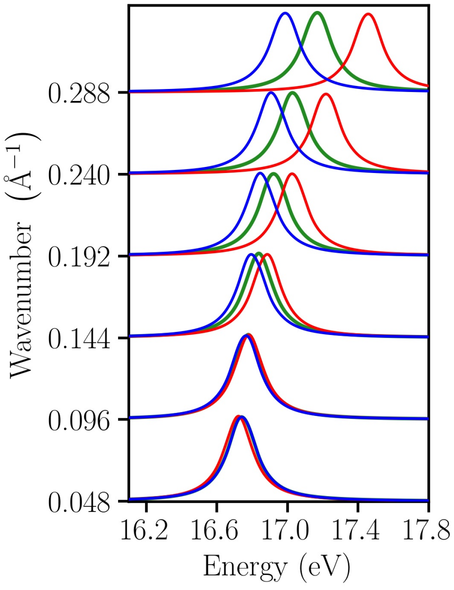

FIG. 5. The EELF in transverse to $\mathbf{Q}$ direction (along z axis) at $U_0 = 0.1$ and different θ values. Red curve for $\theta = 1.0$; green curve for $\theta = 0.5$; blue curve for $\theta = 0.1$.

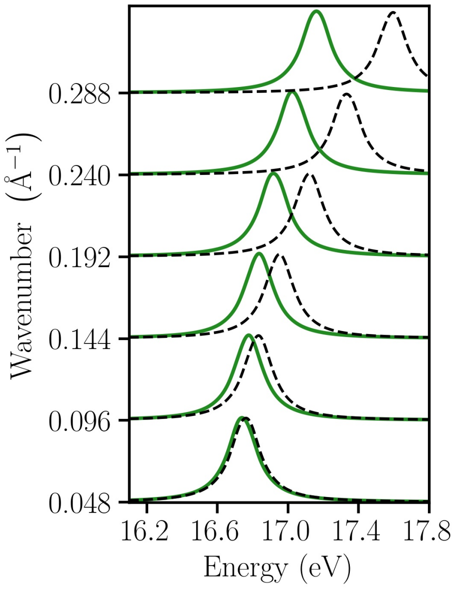

FIG. 6. The EELF at $\theta = 0.5$ and $U_0 = 1.0$. The dashed black curve corresponds to the case of homogeneous system and solid green curve is for the case of inhomogeneous system with $\mathbf{q}$ in transverse to $\mathbf{Q}$ direction (along z axis).

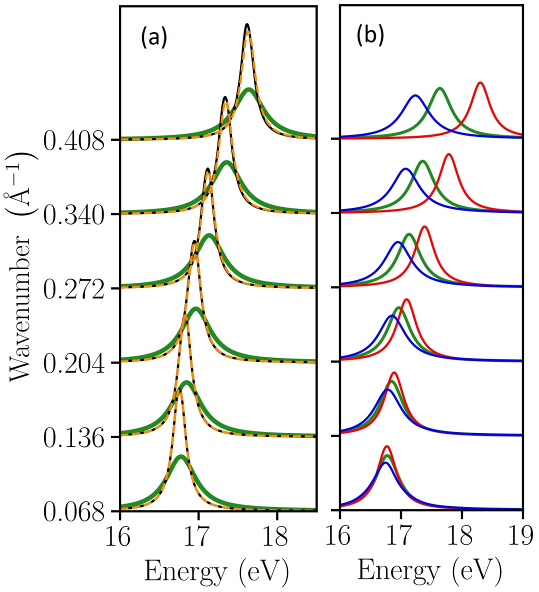

FIG. 8. The EELF at $U_0 = 0.1$. **Left**: The EELF along $\mathbf{q}_{\mathrm{xy}}$(green curve), along $\mathbf{q}_{\mathrm{yz}}$ (black curve), and for the homogeneous system (dashed orange) for $\theta = 0.5$. **Right**: The EELF along $\mathbf{q}_{\mathrm{xy}}$ at different temperatures; red curve for $\theta = 1$; green curve for $\theta = 0.5$; and blue curve for $\theta = 0.1$.

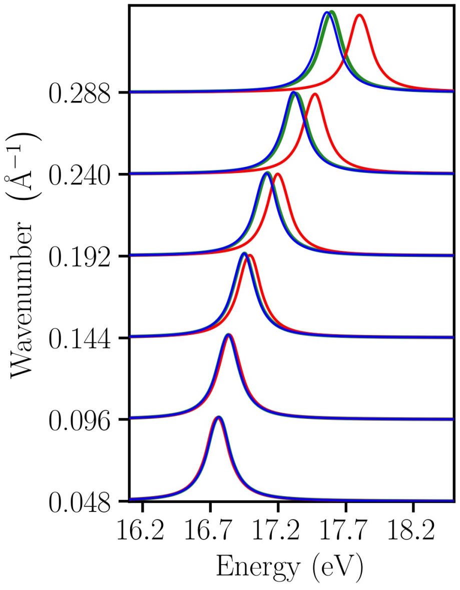

FIG. 7. The EELF in transverse to $\mathbf{Q}$ direction (along z axis) at $U_0 = 1.0$ and different θ values. Red curve for $\theta = 1.0$; green curve for $\theta = 0.5$; blue curve for $\theta = 0.1$.

when the perturbation amplitude is strong $U_0 = 1.0$, the EELF does exhibit a temperature dependence different from the expected variation of the standard plasmon dispersion with increasing q (see Fig. 6).

In the xy and yz planes

Next, we consider the xy and yz planes. Similar to the transverse direction, in Fig. 8(a) we confirm that the EELF of the inhomogeneous system shows a plasmon-like behavior along $\mathbf{q}_{\mathrm{xy}}$ and $\mathbf{q}_{\mathrm{yz}}$. Fig. 8(a) compares the EELF in the inhomogeneous system (orange) with the EELF of the homogeneous system for a perturbation amplitude $U_0 = 0.1$. We see that the dispersion in the oscillations (position of the EELF maximum) in the inhomogeneous system does not deviate significantly from that of the homogeneous system.

Then, we investigate the temperature dependence of these plasmon-like modes. In Fig. 8(b) we illustrate the EELF along $\mathbf{q}_{\mathrm{xy}}$ at different temperatures at a perturbation amplitude $U_0 = 0.1$. While there is a clear temperature dependence at finite q, in the limit $q \to 0$ the sensitivity towards temperature vanishes. This behavior is similar to the temperature dependence expected in the plasmon mode of the homogeneous system.

* a.cangi@hzdr.de

[1] Zhandos Moldabekov, Tobias Dornheim, Maximilian Böhme, Jan Vorberger, and Attila Cangi, "The relevance of electronic perturbations in the warm dense electron gas," The Journal of Chemical Physics, accepted for publication (2021), arXiv eprint 2107.00631.

[2] Tobias Dornheim, Zhandos A Moldabekov, Jan Vorberger, and Simon Groth, "Ab initio path integral monte carlo simulation of the uniform electron gas in the high energy density regime," Plasma Physics and Controlled Fusion **62**, 075003 (2020).

[3] T. Dornheim, J. Vorberger, S. Groth, N. Hoffmann, Zh.A. Moldabekov, and M. Bonitz, "The static local field correction of the warm dense electron gas: An ab initio path integral Monte Carlo study and machine learning representation," J. Chem. Phys **151**, 194104 (2019).

[4] Tobias Dornheim, Jan Vorberger, and Michael Bonitz, "Nonlinear electronic density response in warm dense matter," Phys. Rev. Lett. **125**, 085001 (2020).

[5] Tobias Dornheim, Maximilian Böhme, Zhandos A. Moldabekov, Jan Vorberger, and Michael Bonitz, "Density response of the warm dense electron gas beyond linear response theory: Excitation of harmonics," (2021), arXiv:2104.02405 [physics.plasm-ph].

[6] Tobias Dornheim and Jan Vorberger, "Finite-size effects in the reconstruction of dynamic properties from ab initio path integral monte carlo simulations," Phys. Rev. E **102**, 063301 (2020).

[7] Ethan W. Brown, Bryan K. Clark, Jonathan L. DuBois, and David M. Ceperley, "Path-integral Monte Carlo simulation of the warm dense homogeneous electron gas," Phys. Rev. Lett. **110**, 146405 (2013).

[8] T. Dornheim, S. Groth, T. Sjostrom, F. D. Malone, W. M. C. Foulkes, and M. Bonitz, "Ab initio quantum Monte Carlo simulation of the warm dense electron gas in the thermodynamic limit," Phys. Rev. Lett. **117**, 156403 (2016).

[9] Simone Chiesa, David M. Ceperley, Richard M. Martin, and Markus Holzmann, "Finite-size error in many-body simulations with long-range interactions," Phys. Rev. Lett. **97**, 076404 (2006).
